# Editorial: Neurodegenerative eye diseases: Molecular mechanisms of neurogenesis and therapeutic perspectives

**DOI:** 10.3389/fncel.2022.1060266

**Published:** 2022-10-26

**Authors:** Pooi Ling Mok, Bastion Mae-Lynn Catherine, Suresh Kumar Subbiah, Akon Higuchi

**Affiliations:** ^1^Department of Biomedical Sciences, Faculty of Medicine & Health Sciences, Universiti Putra Malaysia, Seri Kembangan, Malaysia; ^2^Department of Ophthalmology, Faculty of Medicine, Universiti Kebangsaan Malaysia (UKM) Medical Centre, Kuala Lumpur, Malaysia; ^3^Centre for Materials Engineering and Regenerative Medicine, Bharath Institute of Higher Education and Research, Chennai, India; ^4^School of Ophthalmology and Optometry, Eye Hospital, Wenzhou Medical University, Wenzhou, China; ^5^Department of Chemical and Materials Engineering, National Central University, Taoyuan, Taiwan

**Keywords:** neurodegeneration, visual function, regenerative medicine, biomaterials, gene therapy

There are few therapies available for majority of patients suffering from retinal degeneration and other inherited retinal diseases (Higuchi et al., 2017). However, with increasing knowledge of regenerative and developmental neurogenesis to generate retinal tissues and cells, therapeutically effective measures are just on the horizon. Despite this knowledge, only a few clinical trials have been published. This is in part due to the fact that biological mechanisms in the pathogenesis of neurodegeneration in ocular diseases, and how these molecular mechanisms vary with an interventional treatment are still unknown. This Research Topic focusses on the facilitation of neurogenesis for treatment of visual dysfunction.

Optic neuritis and demyelination of the optic nerve are the typical clinical features of multiple sclerosis, which causes patients to suffer persistent visual symptoms because of secondary retinal ganglion cell (RGC) death, as well as optic nerve degeneration. The mouse experimental autoimmune encephalomyelitis (EAE) model models axon loss, extensive RGC soma, and replicates optic neuritis. Nicotinamide mononucleotide adenylyltransferase (NMNAT) is a NAD^+^ (Nicotinamide adenine dinucleotide)-synthetic enzyme, that is critical for axon integrity, and activation of which extensively delays axonal Wallerian degeneration. NMNAT2 that is found in axons, is suggested as a reasonable therapeutic target for neurodegeneration induced by axonal injury. Hence, Liu et al. studied whether activated NMNAT2 could be utilized as a gene therapy scheme for neuronal protection in EAE/optic neuritis. They utilized a RGC-specific promoter to make the expression of the NMNAT2 mutant in mice RGCs *in vivo*, to eliminate the confounding effects in inflammatory cells, which perform important tasks in EAE progression and initiation. However, retina imaging utilizing optical coherence tomography (OCT) *in vivo* suggested no extensive protection of the ganglion cell complexes. The optokinetic response, pattern electroretinography, and visual function assays also indicated no improvement in mouse with overexpressed NMNAT2. Postmortem results of the histological assay of retinal wholemounts and semithin sections of optic nerve indicated that NMNAT2 activation in RGCs provided no extensive neuroprotection in EAE/optic neuritis *in vivo*. Their investigations indicated that a different degeneration mechanism from Wallerian degeneration is observed in autoimmune inflammatory axonopathy, and that NMNAT2 is not the main molecule involved in this regulation.

Chronic kidney disease (CKD) is a critical public health problem because the patient number is rising with the risk of progression to end-stage renal diseases. Examination of the retinal micro-vasculature provides a unique index to evaluate the state of systemic microcirculation. Optical coherence (OCT) angiography (OCTA) parameters provide a noninvasive analysis of the ocular circulation for systemic correlation. Therefore, Yong et al. studied the association of OCTA indices in several causes of CKD. When CKD eyes are compared with a control group for vascular density (VD) and perfusion density (PD), there existed extensive differences between the diabetes mellitus (DM) and control group, but no significant difference was found when compared with the hypertensive-control group or the autoimmune-related glomerulonephritis-control group. There was an extensive correlation between FBS (fasting blood sugar), HbA1c (glycosylated hemoglobin), and age with PD and VD. However, they found no extensive relationship between CKD profile and FAZ (foveal avascular zone). This research highlights the detrimental effects of diabetes mellitus on the vascular density and perfusion of the CKD eye.

The processing pathway of amyloid precursor protein (APP) is altered in Alzheimer's disease (AD), and leads to production of abnormal amyloid-beta (Ab) proteins that generate an insoluble aggregation of interneuron proteins, which are recognized as amyloid plaques in brains. Targeting the pathway of APP processing is important for AD modifying therapies. The protective effects of vitamin E as a signaling molecule as well as an antioxidant are supported by previous research. Therefore, Arrozi et al. studied the modulatory effects of several tocopherol isomers on gene expression served in controlling the pathway of APP processing. Screening of promising tocopherol isomers for their ability to suppress Aβ-42 production and APP expression was performed in SH-SY5Y cells stably overexpressing APP Swedish. Subsequently, quantitative one-step real-time PCR was done to evaluate the modulatory effects of specific tocopherol isomers on gene expression in SH-SY5Y cells overexpressing 3 types of APP (APP Swedish/Indiana, APP Swedish, and wild-type). Their results indicate that every tocopherol isomer, especially at higher concentrations (around 100 mM), extensively enhanced the cell viability in all cell groups, but only γ-tocopherol (GTF) or α-tocopherol (ATF) extensively reduced the APP mRNA expression. Furthermore, GTF and β-tocopherol (BTF) had no influence on Aβ-42 production and the level of APP expression. Results of the study showed that GTF and ATF extensively contributed to a decrease in the expression of Nicastrin, APH1B, and gene beta-site APP cleaving enzyme, but extensively enhanced the expression of Sirtuin 1 in SH-SY5Y cells expressing the mutant APP form. This evidence indicated that GTF and ATF regulates altered pathways and may ameliorate the amyloid burden in AD.

Thymoquinone is a natural occurring molecule, which is the main component of *Nigella sativa*, better known as black cumin. Thymoquinone is commonly used in the Middle East for the treatment of diabetes, hypertension and various other ailments. Beneficial effects of thymoquinone typically originate from its anti-inflammatory, antibacterial, antioxidant, and neuroprotective properties. Currently, there is extensive interest in thymoquinone as a drug for neuronal degeneration in the brain, such as Parkinson's diseases (PD) and AD. Research on animal models of PD and AD suggest that the main neuronal protective mechanisms of thymoquinone originate from its anti-oxidative and anti-inflammatory characteristics. Neuronal degenerative conditions of the eye, such as glaucoma or age-related macular degeneration (AMD) at least in part, have similar mechanisms of neuronal cell death as those found in PD or AD. Therefore, Mahmud N. M. et al. critically analyzed the potential neuroprotective actions and characteristics of thymoquinone for prevention of ocular neuronal degeneration.

Myopia or short-sightedness is a refractive error reaching pandemic levels especially in East Asian countries. Mahmud M. et al. investigated the correlation between visual acuity, and choroidal thickness or foveal photoreceptor layer thickness in highly myopic eyes to evaluate the reasons for reduced visual acuity in the absence of macular pathology. They reported that axial length, age, and foveal photoreceptor thickness (FPT) were moderate predictive parameters for poorer visual acuity in highly myopic eyes with no maculopathy. However, thinner subfoveal choroidal thickness (SFCT) did not result in poorer vision.

This collection of articles highlights the importance of understanding basic disease processes and applying them into clinical situations in the understanding and therapy of ocular degenerative diseases. The eye as an extension of the brain shares many similarities in its function and disease processes. The conclusions of the articles of this special feature may add to the growing literature that may one day lead to eradication of blindness from neurodegenerative processes of the eye.

## Author contributions

PM edited the article. BC and SS advised the content. AH designed and wrote the article. All authors contributed to the article and approved the submitted version.

## Conflict of interest

The authors declare that the research was conducted in the absence of any commercial or financial relationships that could be construed as a potential conflict of interest.

## Publisher's note

All claims expressed in this article are solely those of the authors and do not necessarily represent those of their affiliated organizations, or those of the publisher, the editors and the reviewers. Any product that may be evaluated in this article, or claim that may be made by its manufacturer, is not guaranteed or endorsed by the publisher.
